# The Role of Sexual Prejudice and Aetiology Beliefs in the Italians’ Attitudes Toward Adoption by Same-Sex Couples

**DOI:** 10.5964/ejop.7243

**Published:** 2022-11-30

**Authors:** Silvia Di Battista, Daniele Paolini, Lucia Mongelli, Monica Pivetti

**Affiliations:** 1 University of Bergamo, Bergamo, Italy; 2 University IUL, Florence, Italy; 3 University of Chieti–Pescara, Chieti, Italy; Open University, Milton Keynes, United Kingdom

**Keywords:** aetiology beliefs, sexual orientation, sexual prejudice, same-sex couples, same-sex parenting, gay fathers, lesbian mothers

## Abstract

Research found that those who believe sexual orientation is inborn have generally positive attitudes toward gay men and lesbian women. However, other studies have also found that these beliefs could include negative eugenic ideas. This study aims to investigate the role of people’s beliefs about the aetiology of sexual orientation in attitudes toward adoption for both gay and lesbian couples in Italy. We hypothesized that this relationship would be mediated by sexual prejudice. To test the predictions, 256 Italian heterosexual participants were asked to answer questions on a scale about their beliefs regarding the aetiology of sexual orientation, sexual prejudice, and attitudes toward adoption by same-sex couples. Results confirmed that the relationship between aetiology beliefs and support for adoption by gay and lesbian couples was fully mediated by sexual prejudice. These results suggest that the belief that sexual orientation is controllable may serve to justify one’s prejudice and, in turn, result in a lower support for same-sex couples’ adoption.

Opinions of Italian people toward adoption for same-sex couples have rapidly become more positive in recent years. Eurispes (the Italian Institute for Political, Economic, and Social Studies) surveys showed that in 2015 only 27.8% of Italians declared themselves to be in favour of adoption by same-sex couples, while in 2020 this percentage rose to 42% (Eurispes, 2020). In 2016, the Italian Parliament recognized the civil partnerships for same-sex couples. However, reproductive rights for same-sex partners and parenting were excluded. Currently, the medically assisted reproduction for same-sex couples (e.g., use of donated gametes and embryos, surrogate motherhood) is not allowed (Law 40/2004, Art. 5 and Art. 12). Furthermore, the process of recording children born abroad from surrogacy or donor insemination in the national registry of births is complex for Italian same-sex couples. For instance, in April 2020, the Italian Supreme Court (the highest court in Italy) denied a couple of lesbian women the possibility of recognizing jointly a child who was born in Italy but conceived abroad through the use of medically assisted procreation (Resolution 7668/2020). The Supreme Court also denied this possibility to a couple of gay men for their child who was born abroad through surrogacy (Resolution 12193/2019). Furthermore, in Italy, in a limited number of situations, step-child adoption and foster care can be done by a court order regulated by the Law No. 184/1983–Art. 44 (the so-called “Adoptions in Particular Cases”). However, adoption is in principle permitted only to couples who must be of different-sex by the Law No. 149/2001 (Art. 6, modifying the Law No. 184/1983). This situation persists although research showed that no differences were found for children raised by same-sex parents as compared with those raised by different-sex parents (e.g., Carone et al., 2018; Fedewa et al., 2015; Patterson, 2017).

Research has revealed that negative attitudes toward same-sex parenting are widespread (e.g., Hosking & Ripper, 2012; Tušl et al., 2020; Webb & Chonody, 2012). Italian people still show a preference for the adoption by different-sex parents than for both lesbian and gay couples (e.g., Di Battista et al., 2020). The most common arguments against same-sex parents are either based on judgments of inability to be parents or on the negative effects that these parents have on child development (Clarke, 2001). People who hold more negative attitudes toward same-sex parenting are more likely to be heterosexual men than heterosexual women (e.g., Costa et al., 2019; Rye & Meaney, 2010; Smith et al., 2011), and to be fervent adherents of their religious faith (e.g., Crawford et al., 1999; Costa et al., 2019). Heterosexual men endorse higher levels of sexual prejudice than heterosexual women in many countries and cultures (Ciocca et al., 2015; Furnham & Saito, 2009; Harbaugh & Lindsey, 2015; Herek, 2002; Parrott et al., 2002) including Italy (Ciocca et al., 2015; Lingiardi et al., 2016). Another key factor in predicting negative attitudes toward same-sex parenting is what people think about the controllability of sexual orientation and the developmental origins of non-heterosexual behaviors (e.g., Costa et al., 2019; Crawford et al., 1999; Frias-Navarro et al., 2015; Rye & Meaney, 2010; Smith et al., 2011). Indeed, perceiving controllability for non-heterosexual behaviors suggests a level of choice which is sometimes accompanied by negative evaluations of same-sex parent families (e.g., Costa et al., 2019; Crawford et al., 1999).

In this study, we argued that belief that sexual orientation is controllable may serve to justify negative attitudes in those with high levels of sexual prejudice and subsequently result in opposition to adoption by same-sex couples. In Italy, these negative beliefs would be institutionally reinforced by religious preaching and would be more likely to remain undisputed among heterosexual men (e.g., Ciocca et al., 2015; Lingiardi et al., 2016; Scandurra et al, 2017).

## The Beliefs on the Aetiology of Sexual Orientation

Sexual orientation can be seen as either controllable, thus changeable (i.e. influenced by social and environmental factors, or by individual choice), or not controllable and thus unchangeable (i.e. influenced by biological or genetic factors). In literature, perceiving controllability for sexual orientation is associated with negative evaluations of same-sex behaviors and relationships (Costa et al., 2019; Whitley, 1990). Haslam and Levy (2006) argued that the belief that same-sex sexual orientation is biologically based, immutable, and fixed early in life is one dimension of the essentialist beliefs. In general, essentialist beliefs are a set of ontological assumptions that have important implications for attitudes and they involve an inappropriate understanding of social categories such as “natural kinds”. Following the study by Rothbart and Taylor (1992), Haslam and Levy (2006) argued that essentialist beliefs are a fundamental component of prejudice, because they tend to accentuate group differences. For instance, studies found that the belief in the biological basis of gender and race is associated with greater endorsement of gender and racial stereotypes (Martin & Parker, 1995). However, Haslam and Levy (2006) showed that some essentialist beliefs applied to sexual orientation, particularly the belief that same-sex sexual orientation is biologically based and immutable were not associated with negative attitudes but with positive ones. In their investigations, participants who expressed stronger anti-gay attitudes saw being gay or lesbian as more changeable and culturally specific, rather than being biologically based.

In general, research has consistently found that those who believe sexual orientation is inborn or based on biological factors (such as genetics) and that it is not a personal choice, are more likely to have positive attitudes toward gay men and lesbian women (Haslam et al., 2002; Hegarty, 2002; Hegarty & Pratto, 2001; Smith et al., 2011). On the contrary, heterosexual people who perceive same-sex behaviour as a choice or a controllable state were found to hold higher levels of sexual prejudice (e.g., Costa et al., 2019; Hans et al., 2012). For instance, Whitley (1990) examined how college students’ perceptions of the controllability of being gay or lesbian influence their perceptions of gay men and lesbian women. Results of this study showed that heterosexual individuals’ attitudes were more negative when same-sex sexual orientation was attributed to controllable than to uncontrollable causes. However, the authors cannot confirm the prediction that people expressing more favourable attitudes toward lesbian and gay people attribute same-sex sexual orientation to less controllable causes. Investigating predictors of sexual prejudice regarding lesbian and gay parenting among a sample of Portuguese heterosexual people, Costa et al. (2019) revealed that being older, male, and more religious, as well as having a lower education level, were associated with stronger beliefs of same-sex sexual orientation as being controllable, and these beliefs were in turn associated with more negative attitudes toward same-sex parenting. Exploring Portuguese university students’ attitudes toward same-sex parenting and toward gay and lesbian rights, Costa et al. (2014) also revealed that men were significantly more likely than women to believe that “Homosexuality is a choice”, that “Parents play an important role in the development of their children’s sexual orientation”, that “Homosexuality is learned in contact with homosexual people”, that “Homosexuality is a mental illness”, and to disagree that “Homosexuality is as natural as heterosexuality” (p. 1677). Gender differences were also found in relation to support for gay and lesbian civil rights: Men were more likely than women to be uncomfortable with same-sex marriage and to disagree with the need for gay and lesbian people to fight for their rights and, in particular, the right to have children. Authors also found that beliefs about the social and environmental basis of same-sex sexual orientation were weakly correlated with negative attitudes toward same-sex parenting and toward gay and lesbian civil rights, while the belief that same-sex sexual orientation is a mental illness was highly correlated with these attitudes. These authors concluded that the beliefs about the aetiology of same-sex sexual orientation “may not be direct predictors of attitudes toward gay and lesbian rights, but more closely linked to negative affective reactions toward lesbian and gay men, i.e., homophobia” (p. 1681). In a sample of Spanish university students, Frias-Navarro et al. (2015) found that when participants attributed a non-controllable origin to the same-sex sexual orientation, they showed greater support for the rights of gay men and lesbian women to marry and adopt children, compared to the participants who believed that the origin was environmental. Rye and Meaney (2010) investigated the predictors of Canadian psychology students’ attitudes toward same-sex parents’ adoption using a quasi-experimental design in which participants read one of three adoption scenarios (i.e., heterosexual couple, gay male couple, or lesbian couple). Results showed that beliefs about the aetiology of same-sex sexual orientation were predictive for both men’s and women’s attitudes: Participants who more strongly endorsed environmental explanations of same-sex sexual orientation rated same-sex parent adoption candidates more negatively than those who endorsed biological explanations. Investigating the attitudes toward adoption by gay and lesbian couples in a sample of Psychologists across the United States, Crawford et al. (1999) found that beliefs about the aetiology of same-sex sexual orientation was the most predictive variable in the custody recommendations for same-sex partners. Participants who believed same-sex sexual orientation is a matter of choice were more likely not to recommend custody for both gay and lesbian couples. Smith et al. (2011) explored the relationships between beliefs about the aetiology of same-sex sexual orientation, sexual prejudice, and support for gay-relevant legislation (comprising adoption) among U.S. undergraduate psychology students. Results revealed that the relationship between aetiology beliefs and support for gay-relevant legislation was mediated by sexual prejudice. The authors proposed that it is not the existence of empirical evidence for either side of the nature versus nurture aetiology debate on sexual orientation that affects the negative attitudes toward gay- and lesbian- relevant legislation, but rather how people use their own understanding of the aetiology of sexual orientation. In the same way, Haslam and Levy (2006) also suggested that the associations between the beliefs about the aetiology of sexual orientation and prejudice or discrimination are not straightforward. Other studies have also found that the belief that same-sex sexual orientation is based on biological factors could also increase sexual prejudice and discriminatory policies (Halley, 1994; Haslam et al., 2004), because it can be used to support negative opinions, including those of people who agree with negative eugenic ideas (Sheldon et al., 2007). Furthermore, Haslam et al. (2002) found that even if beliefs in the immutability and naturalness of male same-sex sexual orientation were associated with less negative attitudes toward gay men, essentialist beliefs were also associated with the perception of gay men as being a categorically different “species”, a belief that may increase intolerance. In Croatia, Huic et al. (2018) investigated essentialist beliefs about same-sex sexual orientation as determinants of discriminatory intentions against gay men and lesbian women, and the readiness to engage in positive action toward them. Results showed that essentialist beliefs about homosexuality were strong predictors of both negative and positive behavioral intentions. Beliefs about the immutability of same-sex sexual orientation were associated with fewer intentions to discriminate, and more readiness to engage in positive behavior, while discreteness beliefs (a belief that there are clear and sharp boundaries between sexualities) were inversely related to both. Furthermore, essentialist beliefs impacted attitudes toward lesbian and gay people which were in turn associated with intention to discriminate, and readiness to engage in positive action.

In this investigation and linked to the aforementioned literature, we intended to explore Italian cisgender heterosexual beliefs about the aetiology of sexual orientation. Particularly, we examined how the aetiology beliefs would affect sexual prejudice toward same-sex parents and people’s acceptance of adoption by both gay and lesbian couples. What is missing in the literature is a more solid understanding of the relationship between the beliefs in the aetiology of same-sex sexual orientation and the negative attitudes toward adoption by both gay men and lesbian women couples in Italy. We proposed that an increase in personal beliefs that same-sex sexual orientation is controllable would be related to higher levels of sexual prejudice toward same-sex parents that, in turn, would impact on negative attitudes toward same-sex parents’ adoption. Therefore, we tested the role of sexual prejudice as a mediator in the relationship between Italians’ aetiology beliefs and their level of support for adoption by both lesbian couples (*Hp1*) and gay couples (*Hp2*). Furthermore, as previous research showed that women were more likely to support same-sex parent adoption and reported sexual prejudice less often than men (e.g., Salvati et al., 2019), one could expect that women would be more in favour of same-sex (step)parenting than men. Furthermore, in Italy, religious institutions might tend to reinforce the belief that same-sex orientation is controllable, and even changeable, increasing negative attitudes toward lesbian women and gay men and same-sex parenting. For these reasons, we controlled for gender and religiosity.

## Method

### Participants and Procedures

This empirical study consisted of a 20-minute self-reported questionnaire implemented online using the Qualtrics.com form and administered throughout Italy. A total of 285 questionnaires were administered online. Since the study aims to investigate Italian heterosexuals’ attitudes toward adoption by same-sex couples, non-heterosexual participants were not included in the analyses (*n* = 29). The final sample consisted of 256 self-identified cisgender and heterosexual Italian men (*n* = 128) and women (*n* = 128) aged from 18 to 72 years (*M* = 36.48 years, *SD* = 13.09). The sample was medium-to-highly educated with 46.5% having completed secondary school (*n* = 119), 45.3% having a university degree (*n* = 116), and 8.2% having a post-graduate degree or a PhD qualification (*n* = 21). Participants originated from regions of the center (*n* = 112; 43.8%), the south (*n* = 105; 41%), and the north (*n* = 39; 15.2%) of Italy. As regards religious belief, 102 participants (39.8%) were not religious at all, 75 were occasionally religious people (29.3%), and 79 were medium-to-highly religious people (30.9%). The research complied with the Ethics Code of the Italian Psychology Association (Associazione Italiana di Psicologia, 2015), and it was conducted in accordance with the World Medical Association's Declaration of Helsinki (1964/2013).

### Measures

#### Aetiology Beliefs

To measure participants’ ideas about the origins of sexual orientation, a measure of beliefs about the controllability of sexual orientation was administered (adapted by Costa et al., 2019; Smith et al., 2011). The instrument measures beliefs about the developmental origins and controllability of same-sex sexual orientation. The six items included the following statements: “*Children raised without clear gender roles are more likely to be gay or lesbian*;” “*Individuals who have more stressors and pressures put on them may become gay or lesbian as a result*;” “*Having a dysfunctional family is a cause of being gay or lesbian*;” “*Being gay or lesbian has biological bases* (R);” “*People are born gay or lesbian* (R);” “*Being gay or lesbian is not a choice* (R).” We assessed each item on a Likert-type scale from 1 (Strongly Disagree) to 9 (Strongly Agree). Three items that indicated a greater belief that sexual orientation is inborn were reverse coded and then, the six items were averaged to form a reliable measure (α = .84). Higher scores on this measure indicated a stronger belief that a same-sex sexual orientation is due to a social reaction to one’s environment and that being gay or lesbian is controllable.

#### Sexual Prejudice Against Same-Sex Parents

Five items of the attitudes toward the gay and lesbian parenting scale (adapted from Costa et al., 2014) measured participants’ beliefs about the negative impact of gay and lesbian parents on child development and whether gay men and lesbian women were unfit parents (“*Gay men and lesbian women should not have children because it is a sin;*” “*Gay and lesbian parents do not care about children’s best interests;*” “*Children of gay and lesbian parents will become gay or lesbian or will be confused about their sexuality;*” “*Children of gay and lesbian parents do not have the needed masculine and feminine references for their normal development*;” “*It is not natural for gay men and lesbian women to have children*.”) We assessed each item on a Likert-type scale from 1 (Strongly Disagree) to 5 (Strongly Agree). Then, the items were averaged to form a reliable measure (α = .91). Higher scores on this measure indicate a greater sexual prejudice.

#### Attitudes Toward Adoption by Gay and Lesbian Couples

Two independent items of the Attitudes toward Pathways to Parenthood for Same-Sex Couples measure (Ioverno et al., 2018) were used to evaluate attitudes toward the adoption pathway to parenthood for both gay couples and lesbian couples. Participants first read: “*How much are you in favour of these methods used for having children by gay men couples (couples made up of two men) or lesbian women (couples made up of two women)?*” They then rated the “*adoption for lesbian couples*” and “*the adoption for gay couples*” on a 7-point scale from 1 (Completely Contrary) to 5 (Completely Favourable). Higher scores on these items indicated positive attitudes toward adoption by gay or lesbian couples.

#### Demographic Questions

We measured the participants’ gender (1 = male; 2 = female; 3 = other), gender identification (1 = I am identified with the assigned biological sex; 2 = I am not identified with the assigned biological sex), age, nationality, self-reported Italian region (1 = Central Italy; 2 = Southern Italy; 3 = Northern Italy), level of education (from 1 = none; up to 7 = post-graduate degree). Participants were also asked about their sexual orientation by answering one item with four alternative responses (1 = heterosexual, 2 = gay men, 3 = lesbian women, 4 = other). We also measured self-assessment of religious belief and practice with a single item (1 = I am not religious at all; 2 = I am slightly religious; 3 = I am quite religious; 4 = I am very religious; 5 = I am extremely religious).

### Analyses

We used the Statistical Package for the Social Sciences (SPSS 25.0) to run descriptive, reliability, correlational analyses, Analyses of Variance, and regression analyses. In order to test mediational hypotheses, we performed mediation analyses by using the SPSS macro developed by Hayes and Preacher (2014).

## Results

Table 1 shows the means and standard deviations among all variables and the correlations between all measures investigated in the study. The analyses indicated that these measures were related. Significant positive correlations were found between the perception of controllability of same-sex sexual orientation and sexual prejudice against same-sex parents, whereas negative correlations were found between the perception of controllability of same-sex sexual orientation and the support adoption by both gay and lesbian couples. These findings suggested that greater degrees of belief that being lesbian or gay is due to social factors were associated with less support for adoption by same-sex couples. Furthermore, religious belief and practice was found to be positively related with both sexual prejudice and perception of controllability of same-sex sexual orientation but negatively related with the support for adoption.

**Table 1:** Means (Standard Deviation) and Zero-Order Correlations Among Variables

Measure	Means (*SD*)	1	2	3	4
1. Perception of controllability of homosexuality	2.82 (1.83)	1			
2. Sexual prejudice	2.29 (1.25)	.56**	1		
3. Support for adoption by gay couples	4.75 (2.38)	-.45**	-.79**	1	
4. Support for adoption by lesbian couples	4.78 (2.35)	-.45**	-.79**	.97**	1
5. Religious belief	2.00 (0.99)	.21**	.26**	-.45**	-.45**

**p < .001.

In order to investigate the role of gender, one-way Analyses of Variance—ANOVA, were performed. Concerning the perception of controllability of homosexuality, ANOVA yielded a significant main effect of gender, *F*(1, 255) = 15.68, *p *< .001, showing male participants’ perceived homosexuality as more controllable (*M* = 3.26, *SD* = 1.85) compared to female participants (*M* = 2.79, *SD* = 1.71). Concerning sexual prejudice, ANOVA yielded a significant main effect of gender, *F*(1, 255) = 19.01, *p *< .001, showing male participants have higher levels of sexual prejudice (*M* = 2.63, *SD* = 1.26) compared to female participants (*M* = 1.96, *SD* = 1.16). Finally, concerning the support for adoption's paths, we carried out a 2 (*gender*: male or female participants) × 2 (*adoption support*: lesbian couples or gay couples) mixed ANOVA, with the latter factors varying within subjects. The ANOVA did not yield a significant main effect for adoption typology, *F*(1, 254) = .90, *p* = .34, $\eta_{p}^{2}$ = .004, showing no different evaluations concerning the support adoption by gay or lesbian couples (*M*_lesbian_ = 4.78, *SD* = 2.35; *M*_gay_ = 4.75, *SD* = 2.38). However, a reliable main effect for gender, *F*(1, 254) = 14.51, *p *< .001, $\eta_{p}^{2}$ = .05, and a reliable interaction, *F*(1, 254) = 4.01, *p* = .05, $\eta_{p}^{2}$ = .02, emerged showing as follows: (1) Male participants were more supportive for adoption by lesbian couples (*M* = 4.27, *SD* = 2.42) compared to adoption by gay couples (*M* = 4.16, *SD* = 2.46), *F*(1, 254) = 5.36, *p* = .04, $\eta_{p}^{2}$ = .02, while female participants did not differ concerning support for adoption in the case of lesbian or gay candidates (*M_lesbian_* = 5.29, *SD* = 2.18; *M_gay_* = 5.33, *SD* = 2.16), *F*(1, 254) = 0.90, *p* = .34, $\eta_{p}^{2}$ = .004; and (2) female participants were more supportive—compared to male participants—for both lesbian couples candidates, *F*(1, 254) = 12.48, *p *< .001, $\eta_{p}^{2}$ = .05, and gay couples candidates, *F*(1, 254) = 16.18, *p *< .001, $\eta_{p}^{2}$ = .06.

### Mediation Analyses

According to the literature and the rationale described above, we tested two different mediation models (PROCESS Model Number 4) in which aetiology beliefs were modelled as an independent variable, the sexual prejudice against same-sex parents as mediator, and the support for adoption by lesbian couples and by gay couples as separated dependent variables, by controlling for participants’ gender and level of religious belief.

### Support for Adoption by Lesbian Couples

**Figure 1:**
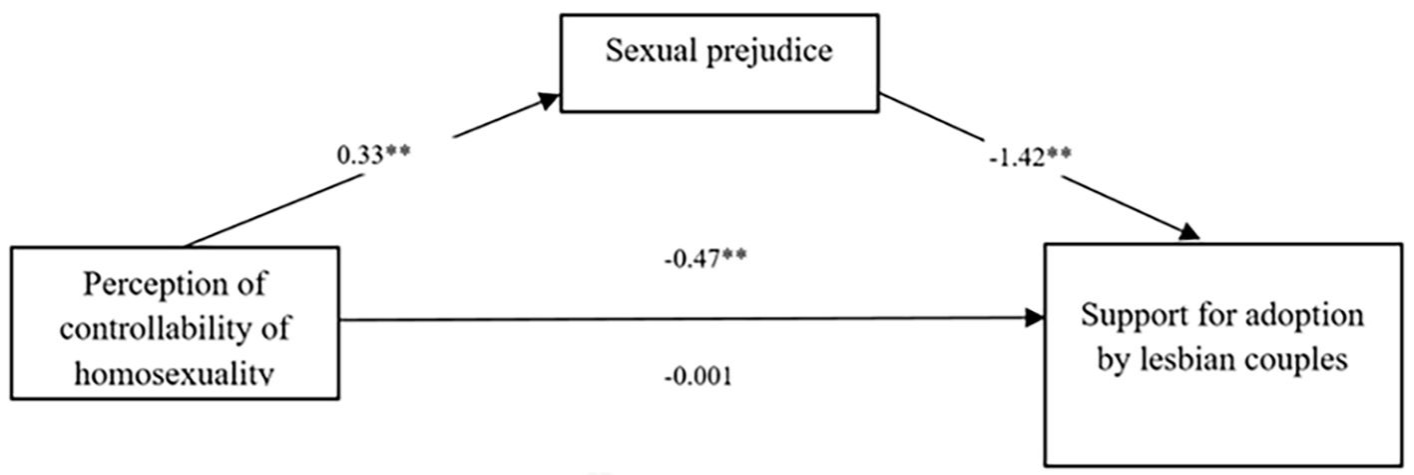
Sexual Prejudice Mediates the Effect of Aetiology Beliefs on Support for Adoption by Lesbian Couples **p < .001.

The model in which the effect of the participants’ aetiology beliefs on adoption support for lesbian couples was mediated by the participants’ level of sexual prejudice was significant, *R^2^* = 0.64; *F*(4, 251) = 112.06, *p* < 0.001 (see Figure 1 and Table 2). Participants’ gender did not emerge as a significant covariate in the full model, *b* = -.12; 95% CI [-0.2469, 0.4938], while the religious belief was significant, *b* = -.27; 95% CI [-0.4630, -0.0910]. The bootstrap analysis with 5,000 resampling showed that indirect effect via the sexual prejudice was significant, *b* = -0.47; 95% CI [-0.6006, -0.3421], while the direct effect considering the mediator was not significant, *b* = - 0.001; 95% CI [-0.1180, 0.1155]. The Sobel test was significant confirming the role of sexual prejudice as a mediator in this model, *z* = 7.86; *SE* = .06*; p *< .001.

**Table 2:** The Results of Mediation Analysis Testing for Lesbian Couples

					95% CI	
Variable	Standardized coefficients	*SE*	*t*	*p*	Lower	Upper
Perception of controllability of homosexuality→Sexual prejudice	.48	.03	9.04	< .001	.2586	.4027
Sexual prejudice→Support for adoption	-.75	.09	-15.97	< .001	-1.5918	-1.2423
Perception of controllability of homosexuality→Support for adoption	-.36	.07	-6.43	< .001	-.6136	-.3260
Indirect effect	-.36	.06	-	-	-.5986	-.3455

*Note*. Covariates: Sex of participants: 0 = male; 1 = female; Religiosity. For indirect effect, the values of *SE* (Standard Error), LLCI (Lower Level of Confidence Interval), ULCI (Upper Level of Confidence Interval) were computed using bootstrapping.

#### Support for Adoption by Gay Couples

**Figure 2:**
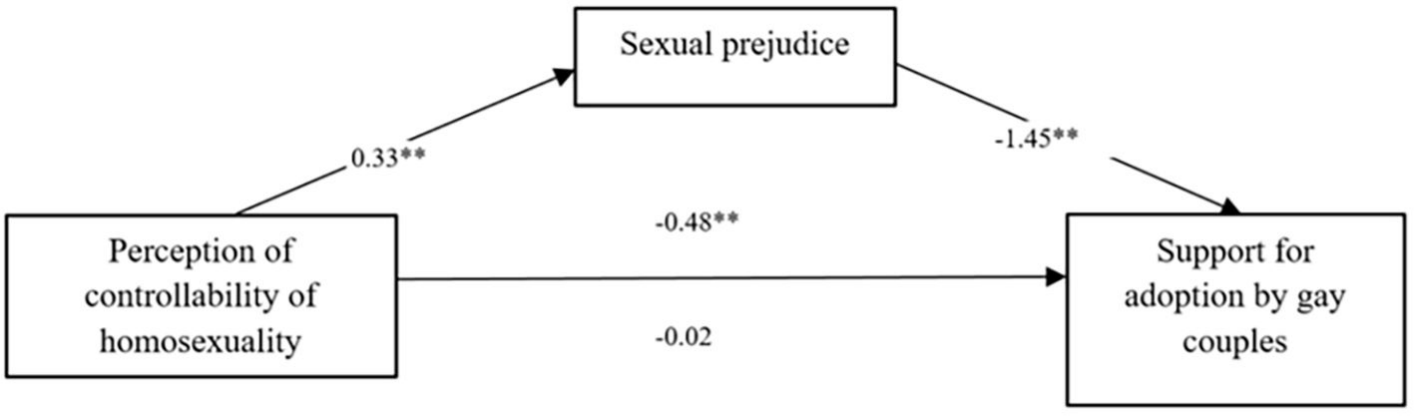
Sexual Prejudice Mediates the Effect of Aetiology Beliefs on Support for Adoption by Gay Couples **p < .001.

The model in which the effect of the participants’ aetiology beliefs on support for adoption by gay couples was mediated by the participants’ level of sexual prejudice was significant, *R^2^* = 064; *F*(4, 251) = 113.78, *p* < 0.001 (see Figure 2 and Table 3). Participants’ gender did not emerge as a significant covariate in the full model, *b* = 0.25; 95% CI [-0.1189, 0.6283], while the religious belief was significant, *b* = - 0.23; 95% [-0.1189, 0.6283]. The bootstrap analysis with 5,000 resampling showed that indirect effect via the sexual prejudice was significant, *b* = -0.48; 95% CI [-0.6123, -0.3530], while the direct effect considering the mediator was not significant, *b* = 0.01; 95% CI [-0.042, 0.1314]. The Sobel test was significant confirming the role of sexual prejudice as a mediator in this model, *z* = 7.89; *SE* = .06*; p* < .001.

**Table 3:** The Results of Mediation Analysis Testing for Gay Couples

					95% CI	
Variables	Standardized coefficients	*SE*	*t*	*p*	Lower	Upper
Perception of controllability of homosexuality→Sexual prejudice	.48	.04	9.04	< .001	.2586	.4027
Sexual prejudice→Support for adoption	-.76	.09	-16.23	< .001	-1.6287	-1.2762
Perception of controllability of homosexuality→Support for adoption	-.37	.07	-6.28	< .001	-.6129	-.3205
Indirect effect	-.37	.05	-	-	-.2559	-.1491

*Note*. Covariates: Sex of participants: 0 = male; 1 = female; Religiosity. For indirect effect, the values of *SE* (Standard Error), LLCI (Lower Level of Confidence Interval), ULCI (Upper Level of Confidence Interval) were computed using bootstrapping.

## Discussion

The present study expands the line of research into beliefs about the aetiology of sexual orientation and sexual prejudice. Results shed more light on the antecedents of support for adoption by gay men/lesbian women and clarified the role of sexual prejudice in modulating the role of beliefs in inborn sexual orientation on support for adoption by gay/lesbian couples. Specifically, the results revealed that cisgender heterosexual Italian participants’ greater beliefs that same-sex sexual orientation is controllable (i.e. due a reaction to one’s social environment) were associated with higher levels of sexual prejudice toward same-sex parenting and less support for adoption by both 1) lesbian and 2) gay parents. Furthermore, the results also revealed that the relationship between aetiology beliefs and support for adoption by both gay and lesbian couples was fully mediated by sexual prejudice toward same-sex parenting. The results suggest that beliefs that sexual orientation is controllable may contribute to one’s prejudice and this prejudice could, in turn, serve to justify the opposition to adoption by same-sex couples. Furthermore, we controlled for gender and religious beliefs which are important predictors of the attitudes toward same-sex parenting in many studies (e.g., Ciocca et al., 2015; Lingiardi et al., 2016). Results showed that Italian men have indeed higher levels of perception of controllability of homosexuality, sexual prejudice, and less tendency to support adoption by same-sex couples (particularly by gay couples) compared to women. However, the gender of participants had no effects in the full mediation models. Religious beliefs did play a role in our model.

These results are consistent with previous evidence that has shown that the perceptions of the controllability of a same-sex sexual orientation are related to more negative perceptions of gay men and lesbian women (Whitley, 1990) and more negative attitudes toward same-sex parenting (Crawford et al., 1999; Frias-Navarro et al., 2015; Rye & Meaney, 2010). Previous studies have also similarly shown that the relationship between aetiology beliefs and support for gay and lesbian people’s rights (including support for adoption by homosexuals) was mediated by sexual prejudice toward lesbian and gay people (Huic et al., 2018; Smith et al., 2011). However, this is the first study to examine how aetiology beliefs and sexual prejudice against same-sex parenting together are negatively related to support for adoption by both lesbian and gay parents in a sample of cisgender heterosexual Italians.

There is scientific evidence and consensus that same-sex parenting does not negatively impact child development (e.g., Hosking & Ripper, 2012; Pistella et al., 2018; Webb & Chonody, 2012). However, sexual prejudice toward both lesbian and gay parents is still widespread throughout Western society. The lack of a social and legal recognition of same-sex parenting is a threat for the psychosocial well-being of same-sex parents and their children (Costa et al., 2019). We point to the need for social policies to reduce prejudice toward sexual minority groups in Italy and in turn to increase the legal recognition of these families. Reproductive rights for same-sex partners and parenting are still excluded in Italy. Furthermore, many Italian conservative and religious people still strongly contest same-sex couples and same-sex parents’ rights (e.g., Baiocco et al., 2020). The biology vs. choice (or nature vs. culture) debate remains a point of serious contention within the LGBT+ community for its possible consequences on sexual prejudice and gay rights (Fry et al., 2020). Within scholarly debates, there are divergent positions on the aetiology of sexual orientation (De Cecco & Elia, 1993). For instance, social constructionists have argued that sexual orientations are a socially constituted entity, whereas essentialists have argued that sexual orientations are natural categories that are grounded in biology. Along these lines, a recent large human genetics study (Ganna et al., 2019) has shown that genetic variation (i.e., the difference in DNA sequences between individuals within a population) accounts only for a small fraction of sexual behavior of gay/lesbian people, leaving room for the many ways in which genetics and the socio-cultural context may interact. However, it is challenging to predict the potential impact which any present or future genetic discoveries will have on the attitudes toward—and the rights of—LGBT+ individuals. We suggest that people with high levels of sexual prejudice could look for any insight that would emerge from scientific debates on the aetiology of sexual orientation to support their discriminatory view. In this line, Haslam et al. (2002) argued that both social constructionist and essentialist positions on the origin of a same-sex sexual orientation can carry risks for sexual prejudice and discrimination. On the one hand, the constructionist belief that sexual orientation is socially determined could justify the acceptance that sexual orientation can be modified ignoring the harm caused by sexual orientation change efforts (APA, 2009). On the other hand, essentialist beliefs on the origin of same-sex sexual orientation can be also used to promote intolerance by those with high levels of sexual prejudice. For instance, Haslam and Levy (2006) argued that biological determinist beliefs represent controversial reasons for challenging antigay attitudes since they can equally be used in the same way to medicalize homosexuality and to promote eugenic ideas. Hegarty (2002) also indicated that the belief in the immutability of sexual orientations does not have a direct impact on tolerance, but it does have one for those people and groups who believe that such an association exists. Therefore, we argue that it is not the existence of scientific evidence for either side of the nature versus environmental aetiology debate that affects discrimination but rather how people perceive or use their own understanding of the aetiology of sexual orientation. Supporting this view, recent experimental studies found that the majority of U.S. people already believe that sexual orientation is not a choice and a scenario including multiple beliefs about sexual orientation aetiology—comprising social constructionist themes—were more effective in reducing heterosexist attitudes toward sexual minorities than focusing only on beliefs about biogenetic origins of sexual orientation (Fry et al., 2020).

In the study, limitations are evident. First, this research is correlational, thus, it is not possible to establish the causality in the relationship between aetiology beliefs and sexual prejudice or support for adoption. However, we assume that aetiology beliefs would precede the other two variables in line with other researches showing a similar pattern of results (e.g., Smith et al., 2011). We have used self-reported measures to measure aetiology beliefs as used in prior research (e.g., Smith et al., 2011). Definitely, the nature of these measures has some criticisms and future studies could propose a quasi-experimental design by manipulating aetiology beliefs. Furthermore, concerning the sample, other demographic variables such as political orientation and/or race/ethnicity of participants may be measured. One limitation of this study is that the sample was disproportionally highly educated and participants mainly came from the regions of central and southern Italy. Since the literature suggested educational level is effective in diminishing sexual prejudice (e.g., Ohlander et al., 2005), further studies should include larger more balanced samples.

Our findings contributed to the theoretical understanding of how the people's support for adoption by same-sex couples is formed. The results can contribute to promote the implementation of more effective and ad-hoc intervention programs to face and fight sexual prejudice suffered by gay and lesbian people.
